# Impacts of streamflow alteration on benthic macroinvertebrates by mini-hydro diversion in Sri Lanka

**DOI:** 10.1038/s41598-020-79576-5

**Published:** 2021-01-12

**Authors:** Dinuke S. N. Munasinghe, Mohamed M. M. Najim, Silvia Quadroni, Muneeb M. Musthafa

**Affiliations:** 1 Department of Geography, University of Alabama, Tuscaloosa, AL 35487 USA; 2 Department of Zoology and Environmental Management, University of Kelaniya, Kelaniya, Sri Lanka; 3 Department of Biosystems Technology, Faculty of Technology, South Eastern University of Sri Lanka, University Park, Oluvil, #32360 Sri Lanka; 4 Department of Theoretical and Applied Sciences, University of Insubria, 21100 Varese, Italy

**Keywords:** Ecology, Environmental sciences, Hydrology

## Abstract

Our study focused on quantifying the alterations of streamflow at a weir site due to the construction of a mini-hydropower plant in the Gurugoda Oya (Sri Lanka), and evaluating the spatial responses of benthic macroinvertebrates to altered flow regime. The HEC–HMS 3.5 model was applied to the Gurugoda Oya sub-catchment to generate streamflows for the time period 1991–2013. Pre-weir flows were compared to post-weir flows with 32 Indicators of Hydrologic Alteration using the range of variability approach (RVA). Concurrently, six study sites were established upstream and downstream of the weir, and benthic macroinvertebrates were sampled monthly from May to November 2013 (during the wet season). The key water physico-chemical parameters were also determined. RVA analysis showed that environmental flow was not maintained below the weir. The mean rate of non-attainment was ~ 45% suggesting a moderate level of hydrologic alteration. Benthic macroinvertebrate communities significantly differed between the study sites located above and below the weir, with a richness reduction due to water diversion. The spatial distribution of zoobenthic fauna was governed by water depth, dissolved oxygen content and volume flow rate. Our work provides first evidence on the effects of small hydropower on river ecosystem in a largely understudied region. Studies like this are important to setting-up adequate e-flows.

## Introduction

The alteration of river flow regimes is claimed to be the most serious and continuing threat to the ecological sustainability of riverine environments^1,2^. The duration and seasonal timing of associated low flow conditions, along with the reduced flow variability, strongly influence riverine organisms directly and via changes to habitat^2,3^. It is thus important to provide means of ensuring future developments that are sustainable and able to protect biological richness and ecosystem functions. This has led to a rapid increase of studies aimed to quantitatively understand aquatic ecosystem responses to various degrees of flow alteration^4,5^.

Small hydropower projects offer one of the most promising energy resources for long-term sustainable development in Sri Lanka. Small hydropower plants have been, and to some extent still are, viewed as an environmentally benign energy source, and are categorized by the Sustainable Energy Authority of Sri Lanka as a green and renewable technology^6^. Small hydropower can, however, exert multiple impacts on local environment (e.g. alteration of flow regime with consequent changes of water physico-chemical parameters and habitat structure), and the impacts which are perceived to be of critical importance are ecological, centered on aquatic flora and fauna. The underlying cause could be attributed to the non-maintenance below the weir of sufficient ‘environmental flow’ (e-flow, i.e. a natural flow paradigm comprising the five components of magnitude, frequency, duration, timing and rate of change), which is recognized as the key to sustaining biodiversity and ecosystem integrity^7^. Previous works on the effects of water depletion on benthic macroinvertebrates in river reaches downstream from diversion structures have frequently pointed out significant impairment, particularly in terms of richness reduction related to habitat trivialization^3^. The impact is higher in river reaches where hydrologic alteration is more severe, i.e. where the entire flow or a very large proportion of it is diverted^8–10^, and where flow alterations cause relevant habitat changes in terms of substrate heterogeneity reduction, nutrient enrichment and temperature regime alteration^11–13^. Biological responses to flow alterations thus strictly depend on the context but also vary according to the kind of off-stream diversion scheme. To date, much of the research has been conducted on dams and impoundments^14–17^, mainly of large size, and few studies have assessed the ecological effects of run-of-the-river schemes^18,19^ and minor intakes^20–22^.

Benthic macroinvertebrates are an important component of the river biota and are indicators of river health^23,24^. There is a wealth of literature suggesting that macroinvertebrate community composition is tightly linked to instream hydraulic conditions^25–27^. The knowledge of associations between environmental factors and zoobenthic assemblages is essential in understanding how aquatic communities in a particular geographic area are structured by the physical and chemical make-up of their environment, how they are affected by alterations to those conditions and, as a consequence, how the health of the entire riverine ecosystem is determined. This knowledge is the base to improve managerial decisions in water resource governance.

A growing need to predict the biological impacts associated with water management activities and to set water management targets that maintain the integrity of riverine ecosystems has created the scientific discipline of ‘in-stream flow’ modelling and design. The primary application of in-stream flow models has been the design of e-flow regimes to guide sustainable water abstractions. Thus, it is evident that decisions on water regulation projects would benefit if they were informed by quantitative predictions of the ecological effects of varying degrees of streamflow alteration. Richter et al.^7,28^ developed and demonstrated the Range of Variability Approach (RVA), a holistic e-flow methodology, for establishing river management targets by incorporating the concept of natural hydrologic variability. RVA accepts that it is not possible to maintain the full range of natural streamflow variability in regulated or otherwise affected river systems, but supports efforts to manage hydraulic alterations in a manner that minimizes impacts on natural hydrologic variability and advocates conservation of native aquatic biodiversity and protection of natural ecosystem functions. In recent years, this approach or a revised version was successfully applied by many authors^29–32^.

Most developing countries still lack the technical and institutional capacity to establish environmental water allocation practices and policies^33^. The existing methods of e-flow assessment are either complex and resource-intensive or not tailor-made for the specific conditions of a country, region or basin^34^. This is the situation in Sri Lanka. In this country, hydroelectricity is the oldest and main source of electricity generation, with a share of nearly 45% of the total available grid capacity in 2010^6^. However, there exists no lawful regulation stipulating the exact quantity of streamflow that should be released as e-flow. In this context, RVA could allow to evaluate the post-diversion circumstances in relation to pre-diversion flows and set management targets to maintain the riverine ecosystems. Since the e-flow concept is relatively new to Sri Lanka, it is also of importance to create awareness among responsible authorities through relevant studies on e-flow assessment.

Therefore, in our study, a holistic e-flow approach for determining streamflow requirements to sustain benthic macroinvertebrate communities and consequently river health below the weir of a small (approx. 5000 kW) hydropower plant in the Gurugoda Oya (a river in Sri Lanka) was explored. Specific aims of the study were (1) to calibrate and validate Hydrologic Engineering Centre—Hydrologic Modelling System (HEC–HMS) 3.5 model and generate long-term flow data for the assessment of post-weir hydrologic alterations, (2) to investigate the spatial variation of water physico-chemical parameters and zoobenthic assemblages above and below the weir, and (3) to identify a-posteriori which physico-chemical parameters are useful to predict the macroinvertebrate community composition through the application of Distance-based Linear Model (DistLM).

We expected that flow abstraction could determine marked changes of key environmental factors which in turn could be associated to biodiversity reduction. We carried out our work during the wet season when the differences due to flow abstraction between impacted and unimpacted sites could be greatest.

## Results and discussion

### Degree of hydrologic alteration due to mini-hydro diversion

**Figure 1:**
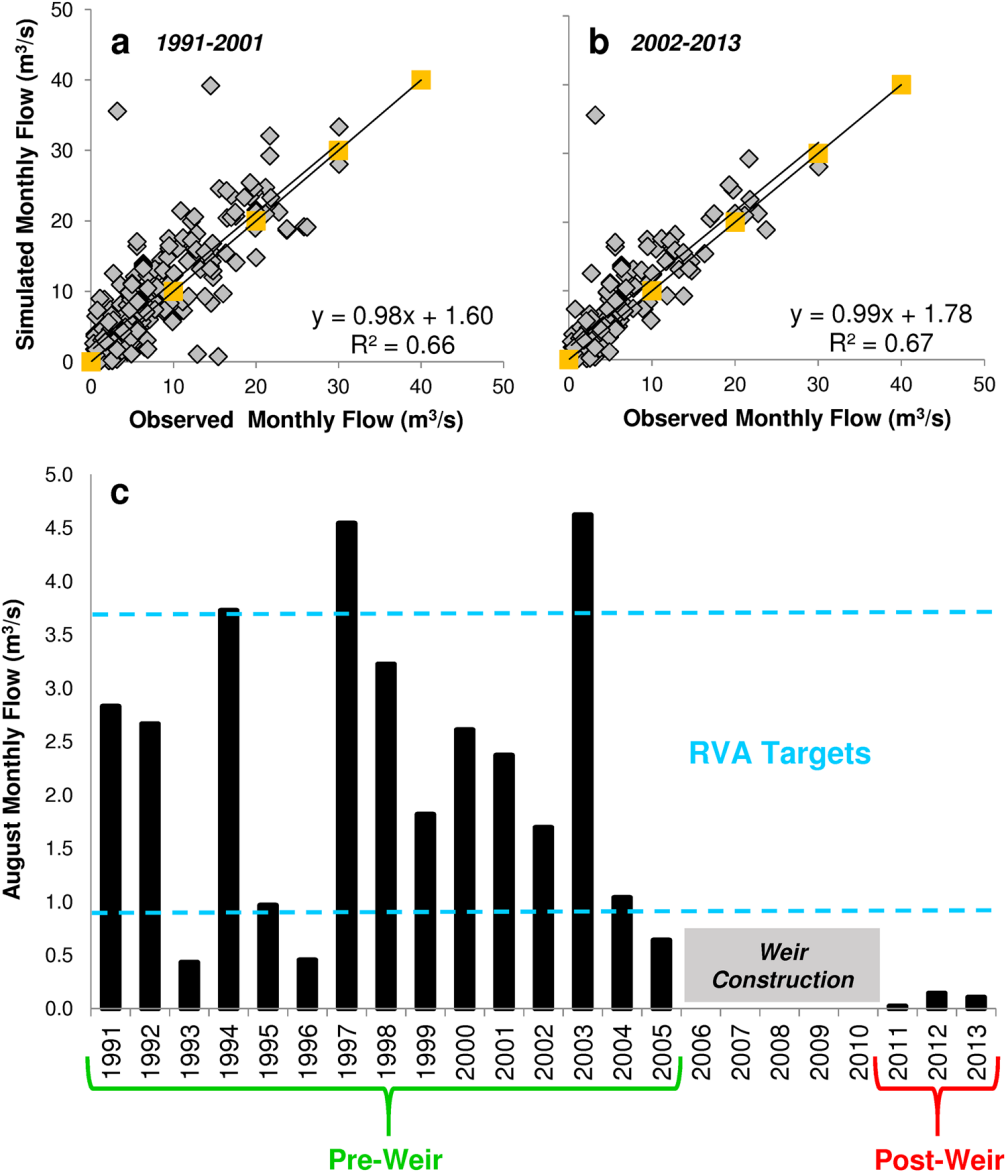
Goodness of fit of simulated (HEC–HMS 3.5 model) and observed (Holombuwa gauging station) flow data, (**a**) model calibration 1991–2001 and (**b**) validation 2002–2013. (**c**) Example of the Range of Variability Approach (RVA) application for the Gurugoda Oya sub-catchment: estimated post-weir values of monthly magnitude of flows in August in comparison to simulated pre-weir flows.

Figure 1a and b show the goodness of fit of simulated flow values against observed flow values for the calibration and validation of HEC–HMS 3.5 model for the Holombuwa catchment. Calibration for the time period 1991–2001 yielded a best scenario of 75.8% of residual points falling within ± 1 SD range, and 96.1% within ± 2 SD range. Moreover, a R^2^ value of 0.66 and a normal distribution of residuals were detected. However, the model slightly over-predicted flows at comparatively lower monthly flow ranges (10–15 m^3^/s). Percentage of residual points within ± 1 SD and ± 2 SD, and R^2^ value of validation results for the next 12 years (2002-2013) were 78.9%, 96.5% and 0.67, respectively. Also the comparison of residuals between observed and simulated flows for the entire study period (1991-2013) yielded results (percentage of residual points within ± 1 SD and ± 2 SD, and R^2^ value of 79.3%, 96.7%, and 0.66, respectively) above the limits suggested by Mood et al*.*^35^, indicating that the validity of the model held even for long periods of runoff simulations in the Holombuwa catchment. The model could thus be successfully applied to the Gurugoda Oya sub-catchment, and RVA targets and rate of non-attainment for the 32 considered Indicators of Hydrologic Alteration (IHAs) were calculated (Fig. 1c and Table 1). Due to the skewness in the distribution of the pre-weir annual values for certain indicators, the mean -1 SD values fell outside (below) the pre-weir low range limits. For these parameters, the pre-weir minima of their range were selected.

**Table 1:** Means and standard deviations of the 32 Indicators of Hydrologic Alteration (IHAs) calculated for the Gurugoda Oya before and after the weir construction. Range of Variability Approach (RVA) targets and rate of non-attainment of the considered indicators are also reported.

		Pre-weir	Post-weir	RVA targets		Rate of non-attainment (%)
		Mean ± SD	Mean ± SD	Low	High	
**Group 1 IHAs (** m^3^**/s)**						
Mean flow of January		1.2 ± 1.0	0.3 ± 0.3	0.2	2.2	33
Mean flow of February		1.4 ± 1.4	0.3 ± 0.3	0.0	2.8	33
Mean flow of March		1.1 ± 1.4	0.5 ± 0.6	0.1	2.5	100
Mean flow of April		2.6 ± 2.2	1.2 ± 1.0	0.4	4.8	33
Mean flow of May		4.3 ± 3.2	1.6 ± 1.5	1.1	7.4	33
Mean flow of June		4.7 ± 3.8	2.3 ± 3.0	0.9	8.5	33
Mean flow of July		2.4 ± 1.8	0.5 ± 0.4	0.6	4.3	33
Mean flow of August		2.2 ± 1.4	0.1 ± 0.1	0.9	3.6	100
Mean flow of September		2.6 ± 2.2	0.3 ± 0.1	0.4	4.8	100
Mean flow of October		6.3 ± 2.3	0.8 ± 0.8	4.0	8.5	100
Mean flow of November		5.4 ± 5.2	3.7 ± 4.8	0.9	10.6	50
Mean flow of December		2.4 ± 2.1	1.0 ± 1.2	0.2	4.5	50
**Group 2 IHAs (** m^3^**/s)**						
1-day minimum flow		0.1 ± 0.0	0.0 ± 0.0	0.0	0.1	33
3-day minimum flow		0.1 ± 0.0	0.0 ± 0.1	0.0	0.1	67
7-day minimum flow		0.1 ± 0.1	0.1 ± 0.1	0.0	0.2	33
30-day minimum flow		0.3 ± 0.3	0.1 ± 0.3	0.0	0.6	67
90-day minimum flow		1.0 ± 0.7	0.4 ± 1.1	0.3	1.7	0
1-day maximum flow		14.8 ± 5.7	5.7 ± 1.3	9.1	20.5	0
3-day maximum flow		13.6 ± 5.1	5.3 ± 1.3	8.5	18.7	0
7-day maximum flow		12.1 ± 4.3	4.8 ± 0.9	7.7	16.4	0
30 day maximum flow		8.2 ± 3.4	3.3 ± 2.1	4.8	11.5	0
90-day maximum flow		5.1 ± 1.7	2.0 ± 0.8	3.4	6.8	0
**Group 3 IHAs**						
Julian date of annual minimum*		127.5 ± 102.4	–	–	–	–
Julian date of annual maximum		101.3 ± 67.5	76.8 ± 87.8	135	169	33
**Group 4 IHAs**						
Low pulse count		90.9 ± 0.3	33.8 ± 0.0	90.7	91.0	100
High pulse count		91.0 ± 0.0	34.0 ± 0.6	91.0	91.0	33
Mean low pulse duration		48.5 ± 20.7	21.9 ± 5.9	27.8	69.3	0
Mean high pulse duration		37.9 ± 12.3	16.9 ± 22.9	25.6	50.2	33
**Group 5 IHAs**						
Mean fall rate		-0.2 ± 0.1	-0.1 ± 0.1	-0.3	-0.1	0
Mean rise rate		0.9 ± 0.2	0.3 ± 0.6	0.7	1.0	100
Fall count		276.4 ± 23.6	91.5 ± 35.8	252.8	300.0	67
Rise count		67.6 ± 8.9	22.4 ± 24.4	58.7	76.5	100

^*^ obtained more than one annual minima

The rate of non-attainment of the group 1 IHAs, which represent the magnitude and timing of flows, was above 30% for the post-weir period. During certain months the rate of non-attainment was 100%. The decrease in the monthly mean flows suggests a drastic drawdown in the water table in the areas downstream of the weir. Group 2 parameters indicated that the daily, weekly, monthly and quarterly minimum flows were negatively influenced by weir regulation. Relevant alterations were also observed in parameters of groups 4 and 5, which represent the timing and frequency, and rate of changes of flow regimes, respectively. Rate of non-attainment of the group 4 IHAs varied between 0 and 100%, and of group 5 IHAs mostly between 67 and 100%.

In summary, out of the 32 IHAs, 11 parameters scored a rate of non-attainment of 33%, and 12 parameters between 50 and 100%. The calculated mean rate of non-attainment of the flow of the Gurugoda Oya below the weir was around 45% (i.e. moderate alteration). Our results proved that the IHA method encasing RVA targets is an easy and useful tool to quantify the hydrologic alteration in the study area as already reported for many rivers worldwide^36–40^. However, these results were preliminary since only three years of data (2011–2013) were used for IHA calculation for the post-weir period. Hence, the high level of fluctuation of rate of non-attainment scored within a single IHA category. It is expected that with time, the fluctuations will decrease and a more reasonable outcome be proposed. However, the implementation of the RVA at an early stage of weir operations could set baselines upon which future river management decisions could be taken and weir operations be performed. For example, the fact that certain parameters scored a rate of non-attainment of 100%, suggests a significant level of hydrologic alteration and calls for immediate changes in weir operations. Thus, the application of RVA to the weir site could be seen as a timely approach to be used as the base for future analyses.

### Effects of weir on water physico-chemistry and benthic macroinvertebrates

A significant difference of the measured physico-chemical parameters (except for pH and five-day Biological Oxygen Demand—BOD_5_) was recorded between study sites upstream (F–E–D) and downstream (C–B–A) of the weir (one-way ANOVA, *p* < 0.05, and Tukey test for pairwise comparison between sites F–E–D and C–B–A, *p* < 0.05). The most prominent feature was the drastic reduction in mean flow velocity and volume flow rate values at the sites below the diversion point (Table 2). It was also evident that, within the sampling period, the volume flow rate above the weir fluctuated heavily whilst below the weir fluctuated within a very narrow range. This shows that the flow released downstream of the weir was heavily regulated irrespective of the rainfall to the locality. Hydropower exploitation frequently induces reduction in streamflow magnitude, which is a strong predictor of biological integrity^41^. However, the impact on aquatic communities is expected to be lower in case of run-of-the-river schemes^18,19^ and minor intakes^20–22^ than in case of reservoirs^14–16^. In the latter case, more severe reductions of the flow variability and changes of flow timing add to the substantial decrease of the flow magnitude.

**Table 2:** Physico-chemical parameters of water measured at the six sampling sites of the Gurugoda Oya (n=7 for each site).

Water parameters	Sites above the weir			Sites below the weir		
	Site F	Site E	Site D	Site C	Site B	Site A
Flow velocity (m/s)	0.19 ± 0.02^d^	0.16 ± 0.02^b,d^	0.11 ± 0.01^b,c^	0.01 ± 0.00^a^	0.05 ± 0.01^a,c^	0.04 ± 0.00^a^
	(0.12–0.29)	(0.10–0.25)	(0.08–0.19)	(0.01–0.02)	(0.03–0.07)	(0.03–0.06)
Temperature (°C)	27.54 ± 0.03^a^	27.54 ± 0.03^a^	27.48 ± 0.04^a^	28.32 ± 0.05^b^	28.29 ± 0.04^b^	27.53 ± 0.10^a^
	(27.42–27.68)	(27.46–27.66)	(27.31–27.65)	(28.12–28.55)	(28.14–28.44)	(27.23–27.85)
Depth (cm)	62.48 ± 3.56^a,b^	66.00 ± 2.45^b^	71.62 ± 2.73^b^	29.33 ± 1.36^c^	50.38 ± 3.16^a^	60.38 ± 5.29^a,b^
	(50.67–78.00)	(57.67–76.33)	(62.00–83.67)	(25.67–37.00)	(39.00–59.33)	(41.33–79.67)
pH	6.98 ± 0.02 ^a^	6.98 ± 0.02 ^a^	6.99 ± 0.02 ^a^	7.00 ± 0.04 ^a^	6.93 ± 0.05 ^a^	6.94 ± 0.05^a^
	(6.91–7.06)	(6.90–7.08)	(6.91–7.06)	(6.81–7.18)	(6.74–7.12)	(6.74–7.07)
Conductivity (µS/cm)	31.26 ± 0.25^b^	31.75 ± 0.21^b^	31.66 ± 0.25^b^	33.35 ± 0.35^a^	33.47 ± 0.16^a^	33.49 ± 0.11^a^
	(30.27–32.23)	(31.23–32.85)	(30.68–32.67)	(31.27–34.13)	(32.85–34.10)	(33.19–34.08)
TDS (mg/L)	20.00 ± 0.00^b^	20.00 ± 0.00^b^	20.14 ± 0.13^b^	21.57 ± 0.27^a^	21.71 ± 0.17^a^	21.86 ± 0.13^a^
	(20.00–20.00)	(20.00–20.00)	(20.00–21.00)	(20.00–22.00)	(21.00–22.00)	(21.00–22.00)
DO (mg/L)	7.46 ± 0.10^d^	7.94 ± 0.03^c^	8.10 ± 0.05^c^	9.77 ± 0.06^b^	8.64 ± 0.04^a^	8.71 ± 0.04^a^
	(7.20–7.80)	(7.80–8.00)	(7.90–8.20)	(9.60–10.00)	(8.50–8.80)	(8.50–8.80)
BOD_5_ (mg/L)	0.93 ± 0.04^a^	0.99 ± 0.05^a^	0.96 ± 0.05^a^	1.04 ± 0.06^a^	1.04 ± 0.06^a^	1.04 ± 0.07^a^
	(0.80–1.10)	(0.80–1.20)	(0.80–1.20)	(0.70–1.20)	(0.80–1.20)	(0.80–1.30)
Volume flow rate (m^3^/s)	4.51 ± 0.97^c^	2.28 ± 0.42^b^	1.49 ± 0.30^a,b^	0.13 ± 0.02^a^	0.72 ± 0.07^a,b^	0.74 ± 0.15^a,b^
	(2.28–9.46)	(1.12–4.45)	(0.87–2.87)	(0.06–0.21)	(0.35–1.00)	(0.27–1.35)

Sites F–D–E are located above the weir, and sites C–B–A below the weir (see Fig. 5). Values are mean ± SD, and range (min-max) within brackets.

TDS, Total Dissolved Solids; DO, Dissolved Oxygen; BOD_5_, five-day Biological Oxygen Demand.

Different superscript letters in a row show significant differences (*p* < 0.05) indicated by Tukey test after one-way ANOVA.

During the sampling period (May-November), 16 benthic macroinvertebrate taxa belonging to three phyla, i.e. Annelida (Oligochaeta), Mollusca (Gastropoda and Bivalvia) and Arthropoda (Malacostraca and Insecta), were found at the six sampling sites (Table 3).

**Table 3:** The overall average composition (AA = absolute abundance—n. individuals, RA = relative abundance—%) and distribution of benthic macroinvertebrate communities in the Gurugoda Oya, above (sites F–E–D) and below (sites C–B–A) the weir (see Fig. 5).

*Taxon*		Sites above the weir						Sites below the weir					
		Site F		Site E		Site D		Site C		Site B		Site A	
		AA	RA	AA	RA	AA	RA	AA	RA	AA	RA	AA	RA
Oligochaeta	*Aeolosoma* sp.	0	0	6	1.9	5	1.2	1	0.7	0	0	2	0.5
Gastropoda	*Pila* sp.	840	75.3	217	68.2	316	76	33	22.2	200	80.6	368	91.3
	*Gyraulus* sp.	117	10.5	0	0	0	0	0	0	0	0	0	0
	*Paludomus* sp.	26	2.3	10	3.1	2	0.5	0	0	0	0	0	0
	*Melanoides* sp.	0	0	0	0	0	0	0	0	6	2.4	0	0
Bivalvia	*Lamellidens* sp.	15	1.3	21	6.6	22	5.3	0	0	0	0	0	0
Amphipoda	*Caridina* sp.	17	1.5	14	4.4	21	5	1	0.7	4	1.6	2	0.5
Decapoda	*Paratelphusa*sp.	23	2.1	7	2.2	11	2.6	0	0	0	0	1	0.2
Coleoptera	*Eubrianax* sp.	0	0	5	1.6	2	0.5	0	0	0	0	1	0.2
Diptera	Tabanid larvae	23	2.1	2	0.6	16	3.8	4	2.7	0	0	0	0
Ephemeroptera	Mayfly nymphs	20	1.8	3	0.9	4	1	17	11.4	21	8.5	15	3.7
Odonata	Damselfly naiads	10	0.9	21	6.6	2	0.5	28	18.8	6	2.4	2	0.5
	Dragonfly naiads	2	0.2	10	3.1	13	3.1	13	8.7	1	0.4	6	1.5
Plecoptera	Stonefly nymphs	3	0.3	0	0	0	0	34	22.8	0	0	0	0
Trichoptera	Caddisfly nymphs	20	1.8	1	0.3	2	0.5	18	12.1	10	4	6	1.5
Hemiptera	*Heleocoris bengalensis*	0	0	1	0.3	0	0	0	0	0	0	0	0
Average total abundance		1116		318		416		149		248		403	

**Figure 2:**
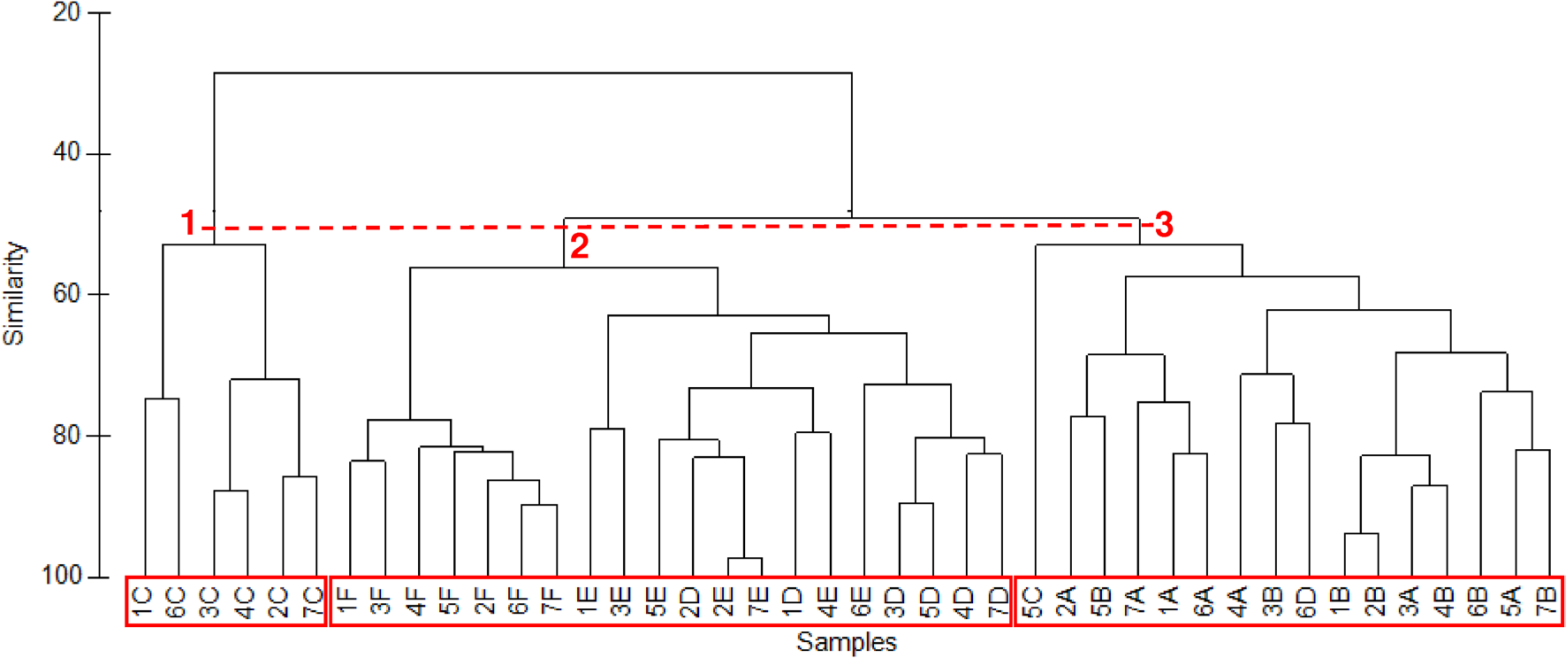
Dendrogram showing the spatial clustering of benthic macroinvertebrate communities between the six study sites (sites F–E–D are located above the weir and sites C–B-A are located below the weir, see Fig. 5) from May (1) to November (7), 2013.

**Figure 3:**
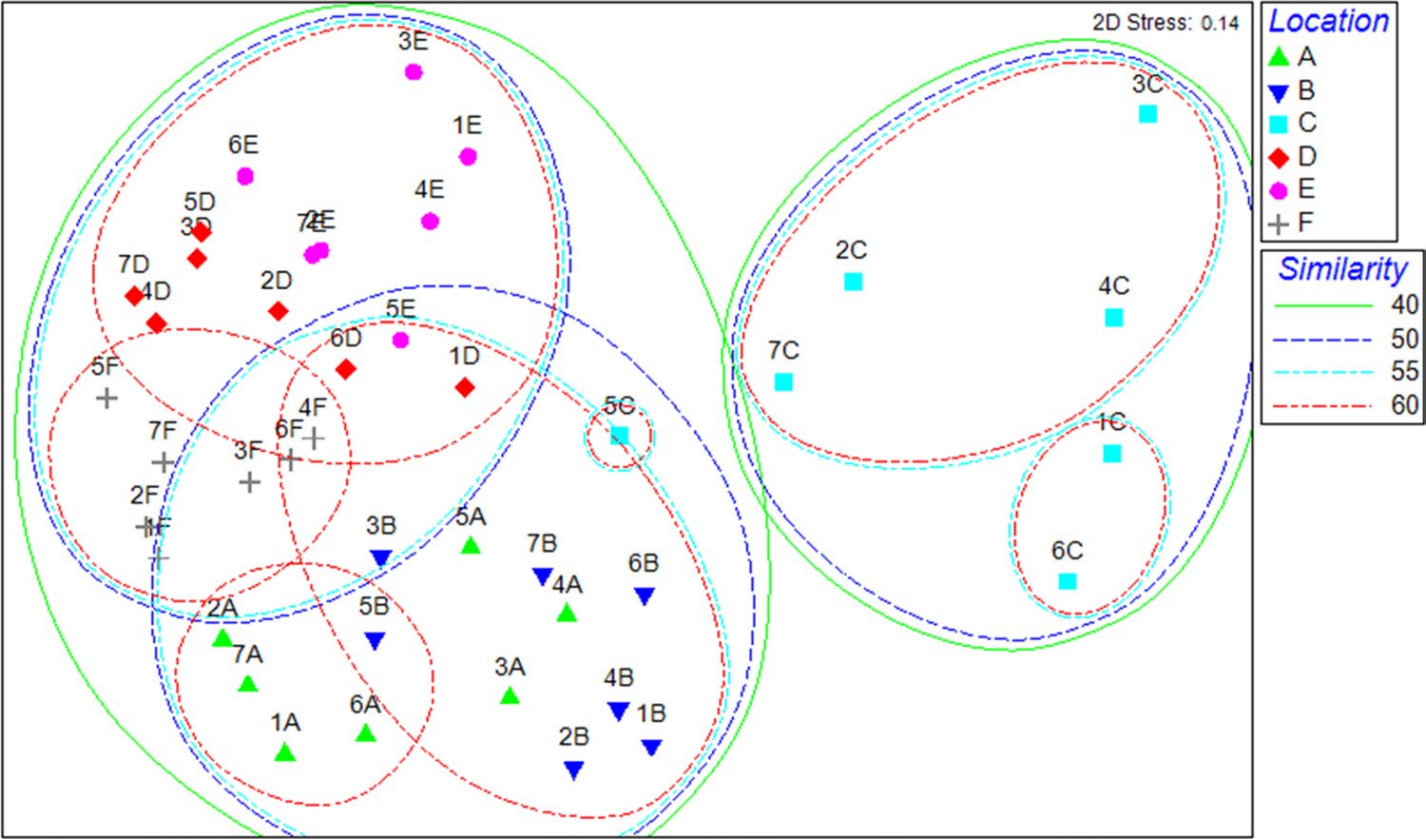
Non-metric Multidimensional Scaling (NMDS) plot depicting the spatial variation of the benthic macroinvertebrate assemblages between the six study sites (sites F–E–D are located above the weir and sites C–B–A are located below the weir, see Fig. 5) from May (1) to November (7), 2013.

Average taxon richness recorded at sites above the weir (12–13) was higher than that at sites below the weir (7–9). However, the cluster analysis detected three major groups at around 50% of similarity (Fig. 2). Sites above the weir differed from sites below the weir, and, among the latter sites, sites A and B differed from site C, i.e. the site closest to the weir (one-way ANOSIM, p<0.05). Also the NMDS plot (Fig. 3) shows how the assemblages collected at site C differed from the assemblages collected at the other sites, with a higher level of intra-site variation between sampling occasions. Moreover, the NMDS plot gives a visual representation of how clustering becomes complex as the similarity level increases. The average abundance of all taxa, except for most of the insect taxa and *Melanoides* sp. were higher at the upstream sites than at the downstream sites. *Gyraulus* sp., *Lamellidens* sp., *Paludomus* sp., and *Heleocoris bengalensis* were even absent at the downstream locations. *Pila* sp. dominated instead all the six study sites except for site C where insect nymphs and larvae displayed similar relative abundances and the lowest average value of total density was observed (Table 3). This pattern was detected by Shannon-Wiener and Pielou’s evenness index, both recording the highest average values at site C (1.86 and 0.85 respectively). Average values highly (0.44 and 0.20) or slightly (0.78 and 0.40) lower than those detected at the three upstream sites (diversity range 1.03–1.31, and evenness range 0.42–0.51) were instead recorded at sites A and B respectively.

**Figure 4:**
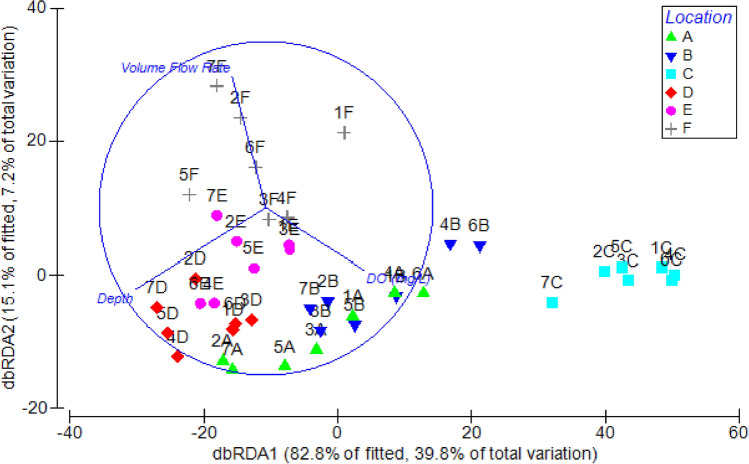
Ordination of the samples based on dbRDA scores of Distance-based Linear Model (DistLM) between physico-chemical parameters (see Table 2) among the six sampling sites (sites F–E–D are located above the weir and sites C–B–A are located below the weir, see Fig. 5).

The analysis of benthic macroinvertebrate communities thus revealed marked dissimilarities between the sites downstream of the weir and the control sites, upstream of the weir. Mini-hydro diversion was associated to taxa loss and consequent richness reduction; only the assemblage detected at site C showed an increase of diversity and evenness. This variable result is common in studies on benthic macroinvertebrate responses to flow alterations. In different contexts, reduced streamflow is reported to induce both decrease and increase in benthos abundance, richness and diversity^15,16,21,42,43^. DistLM (best solution: AICc = 281.9, R^2^ = 0.48, RSS = 27844) identified flow depth, dissolved oxygen (DO) concentration, and volume flow rate (in order of decreasing importance) as the environmental variables driving differences in the structure of macroinvertebrate assemblages of the Gurugoda Oya (Fig. 4), and depicted how different sites are governed by different factors, i.e. site F by the volume flow rate, site D and E by the water depth and site C by the DO content. The dominance of *Pila* sp. may be due to the micro-habitat conditions created by varying flows and effects of drought during the dry season^44^. The major diversity at site C could be attributed to the low depth of the site and the consequent mixing of the water which makes available sediment-bound nutrients into the water column besides increasing oxygen content and micro-habitat diversity. The habitat changes generated by streamflow reduction due to water abstraction can vary widely, depending on channel morphology and substrate stability, and on the possible related nutrient enrichment and temperature regime alteration^11–13,25^. However, the best fit model showed a cumulative percentage variation below 50% on the two major axis (Fig. 4). A major reason for this could be the contribution of other larger-scale environmental or biotic factors on the taxa composition to observed trends in abundance rather than reduced flow. Realistically, it is an interplay of numerous physico-chemical and biotic factors in the hydro-climatic region. For instance, Jayawardana et al*.*^45^ recently highlighted that local riparian forest cover is important in structuring macroinvertebrate communities in the Uma Oya catchment. Also allochthonous nutrient inputs are reported to influence zoobenthic assemblages of Eswathu Oya and Yan Oya^46^. Unfortunately, few other studies on benthic macroinvertebrate communities of Sri Lanka rivers are available^47,48^, and none on the effects of hydropower. Direct comparisons of our data with literature data were thus not feasible, also due to the different resolution level used for taxa identification and sampling methods^49^. To our knowledge, our results provide first evidence for a Sri Lanka river of associations between changes in environmental factors and zoobenthic assemblages consequent to water abstraction due to small hydropower. This is the first step towards the acquisition of the information required to setting-up adequate e-flows^50–53^.

## Conclusions

The HEC–HMS 3.5 model can be endorsed to be reliably used to simulate Gurugoda Oya flows with proper calibration and validation. As the transformation method in the model, ‘snyder unit hydrograph method’ could be recommended for the Gurugoda Oya basin with the ‘initial and constant rate loss’ as the loss method.

The RVA showed that the e-flow was not maintained below the weir throughout 2011-2013. The level of alteration of flows caused by hydropower plant operations was concluded to be moderate (45%). Our results also revealed that the prevailing physico-chemical parameters as well as zoobenthic assemblages varied significantly among the study sites located up- and downstream of the weir. Differences in macroinvertebrate assemblage structure were associated to water depth, dissolved oxygen concentration and volume flow rate.

Although further research is needed to investigate the impact of mini-hydro diversion at a larger spatial and temporal scale, our study should help to pay attention to this relevant and increasing issue in a largely understudied region such as Sri Lanka. We expect that this finding may be used by Government authorities and other policy makers involved in disciplines related to water governance (e.g. Irrigation Department, Sustainable Energy Authority, Ceylon Electricity Board) to make informed decisions on licensing procedures of mini-hydropower plants, and decisions focused on sectoral water allocations for sustainable development while maintaining the integrity of riverine ecosystems.

## Methods

### Study area

**Figure 5:**
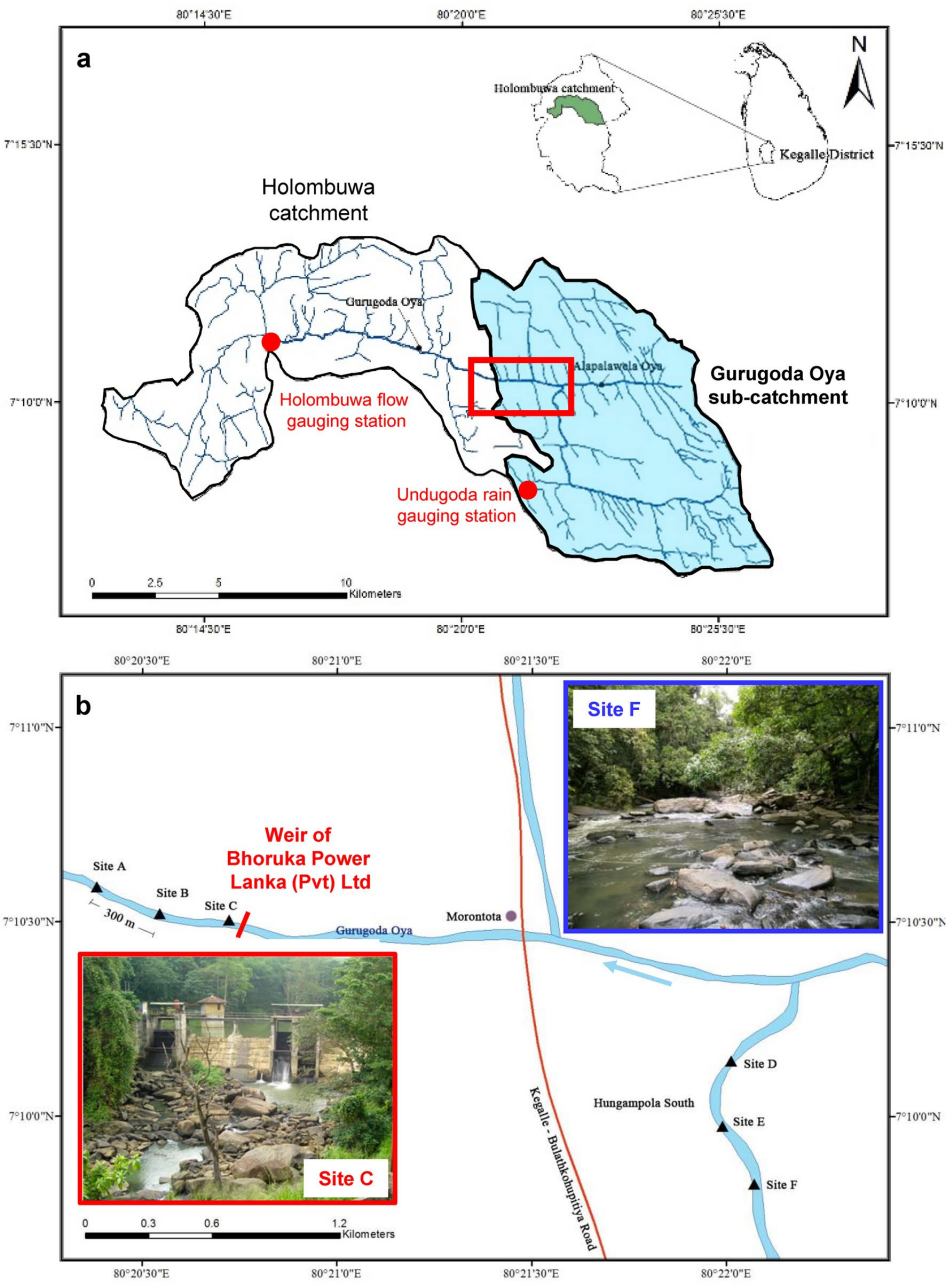
(**a**) Location of Kegalle District in Sri Lanka, and of Holombuwa Catchment in the Kegalle District. The flow and rain gauging station and the Gurugoda Oya sub-catchment (blue) within the Holombuwa catchment are also shown. The red rectangle includes the area where the weir and the sampling sites are located. (**b**) The blow-up shows the six sampling sites with respect to the weir of the mini-hydropower plant in the Gurugoda Oya sub-catchment. The maps were generated using ArcGIS software (version 10.0, https://enterprise.arcgis.com/en/).

The study area is located within Kegalle district in Sri Lanka. It lies entirely within the ‘low country wet’ agro-ecological zone with an expectancy of annual rainfall of 1900–3200 mm^54^. The dry season (rainfall range 50–150 mm) is between November and April, while the wet season (rainfall range 300–500 mm) due to the south-west monsoon between May and October. The mean annual temperature is 27.8 °C and the mean relative humidity is approximately 80%. Rubber plantations and home gardens cover most (74%) of the area. Elevation ranges between 20 and 1240 m asl. Our study was carried out at the Hungampola South/Morontota village areas located mid-east in the Gurugoda Oya sub-catchment which was created within the main Holombuwa catchment (Fig. 5). The reason for the division of a ‘Holombuwa catchment’ and a ‘Gurugoda Oya sub-catchment’ (catchment in relation to the weir site of the mini-hydropower plant) was due to the availability of flow data. Since only the Holombuwa catchment has a flow gauging station, the HEC–HMS 3.5 model was calibrated and validated to this catchment and applied to the Gurugoda Oya sub-catchment to simulate long-term flows at the weir site.

### Model calibration, validation and application

Daily precipitation and monthly evaporation data (1991–2013) at the Undugoda rain gauging station located within the Holombuwa catchment (Fig. 5a), along with daily streamflow data (1991–2013) at the Holombuwa gauging station were obtained from the Hydrology Division of the Department of Irrigation.

For HEC–HMS 3.5 model simulation, the ‘initial and constant rate loss method’ was selected as the loss method and the ‘Snyder unit hydrograph method’ was selected as the transform method based on the model calibration and validation reported by Halwatura and Najim^55^. The Holombuwa catchment was used to accomplish the calibration goal. Daily rainfall data for 11 years (1991–2001) were used in the model and the flows simulated from each calibration run were tested statistically against the actual measured flow to produce a best fit model. The calibrated model was then applied for the period 2002–2013 to accomplish the validation goal and the simulated flows were statistically compared with observed flows for the same time period. Concurrently, a simulation run was also performed with the entire data set (1991–2013), for a further validation. The model parameters were adjusted by certain percentages until the statistical evaluation resulted in more than 68% of the residual points (observed value - simulated value) falling within ± 1SD, more than 95% falling within ± 2 SD and a R^2^ value nearest to 1. According to Mood et al.^35^ a successful model calibration presents the mentioned thresholds. The best parameter distribution scenario was chosen for the subsequent simulation processes. The model was applied to the Gurugoda Oya sub-catchment to generate the daily runoff values for the past 23 years (1991–2013), with the catchment outlet defined as the weir site. The flows which prevailed below the weir (construction period 2006–2010) for the time period 2011–2013 were obtained from data logs of studies conducted in the region and of this study field-sampling campaign.

### Calculation of Indicators of Hydrologic Alteration and rate of non-attainment

The holistic e-flow assessment methodology used in this study is based on the RVA developed by Richter et al.^7^. In the RVA, the pre-weir streamflow regime (1991–2006) was compared with the post-weir flow regime (2011–2013) using 32 ecologically relevant hydrologic parameters (IHAs) (Table 1).

Measures of central tendency (means) and dispersion [range limits (low and high) and standard deviation] were calculated from the pre-weir annual series for each of the 32 parameters, which produced 64 inter-annual statistics for each annual data series (32 measures of central tendency and 32 measures of dispersion), which were used to characterize inter-annual variations. Values at ±1 SD from the mean were selected as the RVA targets (lower and upper RVA limits) for each of the 32 IHAs as recommended by Richter et al.^7^.

The degree of hydrologic alteration or rate of non-attainment of each hydrologic parameter (values that fall below the lower limit and above the upper limit of calculated RVA targets) after the construction of the weir, was then calculated using the following equation^28^:

1 $$ {\text{D}} = \left( {\left| {{\text{N}}_{{\text{o}}} - {\text{N}}_{{\text{e}}} } \right|/{\text{N}}_{{\text{e}}} } \right) \times {1}00 $$

D is the degree of hydrologic alteration/rate of non-attainment, N_o_ is the observed number of post-impact years for which the parameter value falls within the RVA target range, N_e_ is expected number of post-impact years for which the parameter value falls within the RVA target range. N_e_ can be estimated by P x N_T_ where P is the percentage of pre-impact years for which the parameter value falls within the RVA target range and N_T_ is total number of post-impact years. Values between 0 and 33% represent little or no alteration, 33-67% moderate alteration, and 67-100% high alteration. Finally, the degrees of hydrologic alteration of all 32 parameters were averaged to obtain a single level of alteration of flow regime for the weir site. The IHA software (version 7.0) developed by the Nature Conservancy was used to calculate the 32 IHAs, the RVA targets and the levels of alterations of the parameters.

### Collection of benthic macroinvertebrates and water physico-chemical parameters

Six sampling sites were selected in the study area to capture the effects of different flow regimes on benthic macroinvertebrates. Sampling sites F–D–E and C–B–A were established separately 300 m away from each other along the Gurugoda Oya, the former three above and the latter three below the weir (Fig. 5b). The study was carried out from May to November 2013 with monthly intervals between sampling occasions.

At each sampling site, the cross section of the stream was divided into three equal parts and sampling was carried out in the center of these three sections separately. The three replicates were then pooled into one integrated sample. Macroinvertebrates were collected using a standard D-framed dip net consisting of a D-shaped metal frame (0.3 m width and 0.3 m height) holding a conical net (mesh aperture 400 μm). A dip and sweep method was employed where organisms were collected by aggressively disturbing the target habitat. A sweep of 0.5 m length was made per sampling effort. The net was dipped into the substrate and three such sweeps were performed^56^ to collect bottom sediments covering an area of about one square meter^57^. River substrate varied from pebbles to boulders in different percentages with a local presence of soft sediment. The depth of each sampling point was measured using a pole and tape in tandem with benthic sampling. Later in the laboratory, the material retained was wet sieved through a mesh (0.5 mm aperture)^57^ and identified to the nearest possible taxonomic category using the naked eye and a binocular microscope following the standard identification keys provided by Mendis and Fernando^58^ and Starmühlner^59^.

At each sampling site, each time a biological sample was taken, the physico-chemical parameters of the water immediately above the bottom were measured. Such parameters were temperature, pH, conductivity, Total Dissolved Solids (TDS), Dissolved Oxygen (DO), five-day Biological Oxygen Demand (BOD_5_) and water flow velocity. Temperature, pH, conductivity and TDS were measured in situ using the Yellow Springs Instrument (YSI) 55 water quality logger (USA). The DO concentration was measured in the laboratory using the Winkler method^60^, after collecting water samples into amber-colored glass bottles (250 mL) and fixing DO in situ using manganous sulfate and Winkler reagents. Additional water samples were collected into amber-colored glass bottles (250 mL), brought to the laboratory, and incubated for five days at room temperature in total darkness. After the five-day incubation period, DO concentration in each bottle was measured using the Winkler method^60^ and BOD_5_ was determined. The water flow velocity was measured by using a float (a piece of Styrofoam) to drift for a known distance along the water current for a known period of time. Each water parameter was measured in triplicate and the mean value was calculated later on.

### Data analysis

Similarities of macroinvertebrate assemblages were assessed using Bray-Curtis similarity clustering method^61^ on square root transformed abundance data. Moreover, Non-metric Multi-Dimensional Scaling (NMDS)^62^ was performed to better represent the spatial and temporal clustering of the benthic macroinvertebrate communities between the study sites. Analysis of similarities (ANOSIM) was carried out to detect significant differences of community composition among the six sampling sites. Total density, taxon richness, Shannon-Wiener diversity index and Pielou’s evenness index were then calculated for each sample. One-way analysis of variance (ANOVA) and Tukey post-hoc test were used to compare the considered physico-chemical parameters among the six study sites.

Distance-based Linear Model (DistLM)^63^ was applied to perform permutational regression between zoobenthic assemblages and environmental variables assessing the relative contributions of environmental variables structuring macroinvertebrate communities. Prior to DistLM, draftsman plots and correlation matrices were produced to assess the distribution of each variable and to identify co-correlating variables. Environmental variables were square root transformed to normalize their distribution, and since no pairs of variables had a Pearson’s correlation coefficient larger than 0.9, all were included in the analysis. The Bray-Curtis matrix on square root transformed macroinvertebrate abundances was used. DistLM was performed with selection based on the Akaike information criterion (AIC), step-wise selection procedure and 999 permutations. AIC was chosen as the method to create the most parsimonious model, as it adds a ‘penalty’ for increases in the number of predictor variables^63^. Step-wise selection was chosen as it allows for both the addition and removal of a term to the model at each step^63^. Distance-based Redundancy Analysis (dbRDA) plot was used to provide the best possible 2-dimensional visualization of DistLM result.

All the statistical analysis were carried out using PRIMER V 6.1.16 (equipped with PERMANOVA+ V 1.0.6) statistical software.

## Data Availability

Main data generated and analyzed during the current study are included in the manuscript, further information is available from the corresponding author on reasonable request.
